# Trends in Incidence of Early-Onset Colorectal Cancer in the United States Among Those Approaching Screening Age

**DOI:** 10.1001/jamanetworkopen.2019.20407

**Published:** 2020-01-31

**Authors:** Wesal H. Abualkhair, Meijiao Zhou, Dennis Ahnen, Qingzhao Yu, Xiao-Cheng Wu, Jordan J. Karlitz

**Affiliations:** 1 Department of Medicine, School of Medicine, Tulane University, New Orleans, Louisiana; 2 Louisiana Tumor Registry, Department of Epidemiology, Louisiana State University Health Sciences Center, New Orleans; 3 University of Colorado School of Medicine and Gastroenterology of the Rockies, Denver; 4 School of Public Health, Louisiana State University Health Sciences Center, New Orleans; 5 Southeast Louisiana Veterans Health Care System, New Orleans; 6 Division of Gastroenterology, Department of Medicine, Tulane University School of Medicine, New Orleans, Louisiana

## Abstract

**Importance:**

Early-onset colorectal cancer incidence rates among patients aged 45 to 49 years have been considered much lower compared with the rates among patients aged 50 to 54 years, prompting debate about earlier screening benefits at 45 years. However, the observed incidence rates in the Surveillance, Epidemiology, and End Results (SEER) registries may underestimate colorectal cancer case burdens in those younger than 50 years compared with those older than 50 years because average-risk screening is generally not performed to detect preclinical cases of colorectal cancer. Finding steep incidence increases of invasive stage (beyond in situ) cases of colorectal cancer from age 49 to 50 years would be consistent with high rates of preexisting, undetected cancers in younger patients ultimately receiving a diagnosis of colorectal cancer after undergoing screening at 50 years.

**Objective:**

To assess the preclinical burden of colorectal cancer by analyzing its incidence in 1-year age increments, focusing on the transition between ages 49 and 50 years.

**Design, Setting, and Participants:**

Data from the SEER 18 registries, representing 28% of the US population, were used to conduct a cross-sectional study of colorectal cancer incidence rates from January 1, 2000, to December 31, 2015, in 1-year age increments (ages 30-60 years) stratified by US region (South, West, Northeast, and Midwest), sex, race, disease stage, and tumor location. Statistical analysis was conducted from November 1, 2018, to December 15, 2019.

**Main Outcomes and Measures:**

Incidence rates of colorectal cancer.

**Results:**

A total of 170 434 cases of colorectal cancer were analyzed among 165 160 patients (92 247 men [55.9%]; mean [SD] age, 51.6 [6.7] years). Steep increases in the incidence of colorectal cancer in the SEER 18 registries were found from 49 to 50 years of age (46.1% increase: 34.9 [95% CI, 34.1-35.8] to 51.0 [95% CI, 50.0-52.1] per 100 000 population). Steep rate increases from 49 to 50 years of age were also seen in all US regions, men and women, white and black populations, and in colon and rectal cancers. The rate ratio incidence increase in the SEER 18 registries from 49 to 50 years of age (1.46 [95% CI, 1.42-1.51]) was significantly higher than earlier 1-year age transitions. Steep rate increases in the SEER 18 registries were found from 49 to 50 years of age in localized-stage (75.9% increase: 11.2 [95% CI, 10.7-11.7] to 19.7 [95% CI, 19.0-20.3] per 100 000) and regional-stage (30.3% increase: 13.2 [95% CI, 12.7-13.8] to 17.2 [95% CI, 16.7-17.8] per 100 000) colorectal cancers. A total of 8799 of the 9474 cases (92.9%) of colorectal cancer in the SEER 18 registries from 2000 to 2015 that were diagnosed among individuals aged 50 years were invasive.

**Conclusions and Relevance:**

Steep incidence increases between 49 and 50 years of age are consistent with previously undetected colorectal cancers diagnosed via screening uptake at 50 years. These cancers are not reflected in observed rates of colorectal cancer in the SEER registries among individuals younger than 50 years. Hence, using observed incidence rates from 45 to 49 years of age alone to assess potential outcomes of earlier screening may underestimate cancer prevention benefits.

## Key Points

**Question:**

What is the increase in the colorectal cancer incidence rate from 49 to 50 years of age when large segments of the population begin average-risk screening?

**Findings:**

This cross-sectional analysis of colorectal cancer incidence rates in 1-year age increments (30-60 years) from 2000 to 2015 in the Surveillance, Epidemiology, and End Results 18 registries found an incidence rate increase of 46.1% from 49 to 50 years of age. A total of 92.9% of the cases of colorectal cancer diagnosed at 50 years of age were invasive (beyond in situ stage).

**Meaning:**

Steep incidence increases from 49 to 50 years of age are consistent with preexisting colorectal cancers diagnosed via screening uptake, supporting the presence of a large undetected preclinical case burden in patients younger than 50 years that is not reflected in observed Surveillance, Epidemiology, and End Results incidence rates.

## Teaser

This cross-sectional study uses data from the Surveillance, Epidemiology, and End Results registries to assess the preclinical burden of colorectal cancer by analyzing its incidence in 1-year age increments, focusing on the transition between 49 and 50 years of age.

## Introduction

Early-onset colorectal cancer (EOCRC) incidence rates are increasing, and controversy exists regarding whether average-risk screening should begin at 45 or 50 years of age.^1^ In 2018, the American Cancer Society recommended that average-risk screening start at 45 years of age.^2^ Others recommend screening at 50 years of age, although the US Multi-Society Task Force on Colorectal Cancer recommends screening African American individuals at age 45 years of age owing to higher incidence, mortality, and earlier-onset disease.^3,4,5,6^ The American Cancer Society decision incorporated modeling studies that used updated incidence and mortality data encompassing time periods of increasing EOCRC incidence rates; modeling compared life-years gained by initiating screening at 45 vs 50 years.^7,8,9,10^

Aforementioned incidence analyses used EOCRC rates in defined age-group ranges (ie, 40-49 years) stratified over different time periods. However, incidence analysis in 1-year age increments as a continuous variable during a recent time period will allow for the assessment of the transition between the ages of 49 and 50 years. This transition is of particular interest because this is historically when average-risk screening is first recommended.

Generally, owing to a lack of screening, the incidence of colorectal cancer (CRC) among patients 49 years or younger reflects mainly diagnostically detected CRCs (owing to symptoms) or higher-risk screened groups (owing to family histories of cancer), in contrast to those 50 years or older, in whom CRCs are detected owing to both diagnostic testing and average-risk screening. Because many CRCs are asymptomatic, observed incidence rates of EOCRC in the Surveillance, Epidemiology, and End Results (SEER) registries do not reflect preclinical CRC case burdens in younger patients, and we would expect to see some degree of CRC incidence increase from 49 to 50 years of age owing to screening uptake and diagnosis of preexisting CRCs that may have been clinically undetected.^11^ Ideally, any increase in the incidence of CRC would be low, consisting mainly of easily curable in situ lesions. The degree of incidence increase would be expected to be proportional to the preclinical case burden in those several years younger and in their 40s. The degree of such an incidence increase and the CRC stage distributions in those aged 50 years have not been previously reported, to our knowledge. Steep incidence increases from ages 49 to 50 years, consisting mainly of invasive stage (beyond in situ) cases, would be consistent with a high, undetected preclinical EOCRC case burden. Because modeling studies have mainly informed guideline decision-making on screening age determinations (45 vs 50 years), a SEER analysis of this type can provide real-world registry data to help gauge potential outcomes of earlier screening.

Hence, we used data from the 2000-2015 US SEER 18 registries to assess CRC incidence rates in 1-year age increments (30-60 years), focusing on the transition between ages 49 and 50 years. We stratified incidence by geographical region, sex, race, and tumor location (colon or rectum) to determine whether any potential rate increase from 49 to 50 years of age is consistent across different patient groups. We also stratified by cancer stage to assess distributions (in situ, localized, regional, and distant) and determine the percentage of invasive (beyond in situ stage) cases. Finally, we analyzed 5-year relative survival at every age from 30 to 60 years.

## Methods

Data from the January 1, 2000, to December 31, 2015, SEER 18 registries were analyzed. The SEER 18 is a high-quality database with standardized collection practices overseen by the National Cancer Institute, representing 28% of the US population.^12,13^ The SEER data have a case completeness rate greater than 98%.^14^ Data from 2000 to 2015 were used because this range of years encompasses periods of increasing EOCRC rates and the period when screening became commonplace.^15^ These data were the most current data available at the time of analysis. Primary (excluding metastases to the colorectum) CRC cases were included (*International Classification of Diseases for Oncology, Third Edition* codes C18.0-C18.9 and C26.0 for colon cancer and C19.9 and C20.9 for rectal cancer). Only adenocarcinomas were analyzed. The study was reviewed by the Tulane University Biomedical Institutional Review Board and considered exempt given its use of deidentified data. The Strengthening the Reporting of Observational Studies in Epidemiology (STROBE) reporting guidelines for cross-sectional studies were followed.^16^

### Statistical Analysis

Statistical analysis was conducted from November 1, 2018, to December 15, 2019. Annual mean age-adjusted CRC incidence rates per 100 000 population (ie, 2000 US standard population) were calculated using SEER*Stat, version 8.3.5. Rates were calculated at every age from 30 to 60 years and were calculated at the case (tumor) level. Rates were not examined for individuals younger than 30 years owing to low case counts or for those older than 60 years given our goal to understand EOCRC. All *P* values were from 2-sided tests and results were deemed statistically significant at *P* < .05. The Tiwari 95% CI option was selected.^17^

We analyzed the incidence rates in 4 US Census Bureau geographic regions (Southern, Midwestern, Northeastern, and Western).^18^ Sex and race stratification was performed. White and black individuals were analyzed because they are the 2 largest racial groups. Because the incidence of early-onset rectal cancer has increased faster than the incidence of colon cancer, anatomical subsite analysis (rectum and colon) was conducted.^10^

Percentage rate increases and rate ratios were analyzed for 1-year age transitions (47-48, 48-49, and 49-50 years) to determine whether the increase for the transition from 49 to 50 years was highest. Steeper rate ratio inflection points from 49 to 50 years compared with earlier age transitions would support an excess of screening-related cases. We also fitted a log-linear model based on yearly age incidence changes at 30 to 49 years to estimate CRC incidence at 50 years in the theoretical absence of screening (see eFigure 2 in the Supplement for additional details on methods).^19^ The difference between this and the observed rate at 49 years would estimate the rate increase at 49 to 50 years associated with age-related changes alone. Furthermore, a higher observed SEER incidence rate at 50 years compared with what would be estimated at 50 years based on age-related changes alone would estimate the screening-associated increase.

The incidence of CRC was stratified by stage (in situ, localized, regional, and distant) from 30 to 60 years of age.^20^ Stage subgroup analysis (staging information available in 98.1% of cases) was performed based on race and anatomical subsite (rectum and colon).

Yearly-age CRC case counts were generated at each age from 45 to 50 years for SEER 18. Given that SEER 18 represents 28% of the population, to extrapolate and estimate the total number of US cases at each age, a multiplication factor of 3.57 was used.^13^

Finally, we analyzed 5-year relative survival at every age from 30 to 60 years. A significant survival increase from 49 to 50 years would provide support that steep incidence increases from 49 to 50 years may be associated with screening uptake.

## Results

The baseline characteristics of the patients are reported in Table 1. There were 165 160 patients (55.9% men, 75.4% white, and 14.3% black, with a mean [SD] age of 51.6 [6.7] years) representing 170 434 cases of CRC.

**Table 1.** Demographic Characteristics of Participants in the SEER 18 Registries Aged 30 to 60 Years

Characteristic	Participants, No. (%) (N = 165 160)
Age at diagnosis, mean (SD), y	51.6 (6.7)
Sex	
Male	92 247 (55.9)
Female	72 913 (44.1)
Summary stage 2000 (1998 onward)^a^	
In situ	8494 (5.1)
Localized	56 349 (34.1)
Regional	60 196 (36.4)
Distant	36 814 (22.3)
Unknown or unstaged	3307 (2.0)
Race	
White	124 528 (75.4)
Black	23 693 (14.3)
Other (American Indian, Alaskan Native, or Asian and Pacific Islander)	15 452 (9.4)
Unknown	1487 (0.9)

Abbreviation: Surveillance, Epidemiology, and End Results.

^a^ The 2000 version of summary stage, applied to cases diagnosed January 1, 1998, and forward.

**Figure 1.**
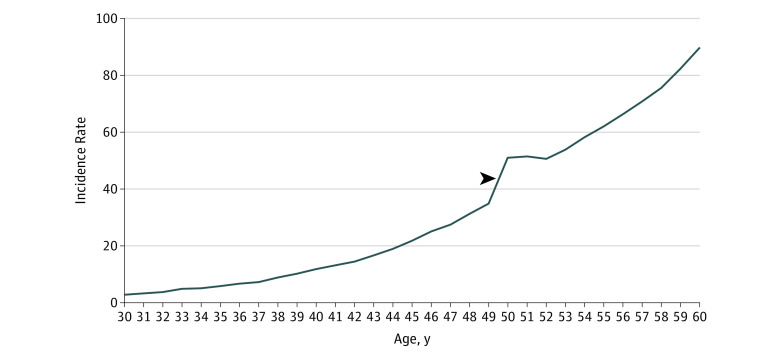
Colorectal Cancer Incidence Rates per 100 000 Population in 1-Year Age Increments in the US Surveillance, Epidemiology, and End Results 18 Registries Among Patients Aged 30 to 60 Years, 2000-2015 Only adenocarcinomas were analyzed. The arrowhead indicates the incidence rate increase from 49 to 50 years of age (46.1% increase: 34.9 [95% CI, 34.1-35.8] to 51.0 [95% CI, 50.0-52.1] per 100 000 population).

Steep incidence increases existed from 49 to 50 years of age in the combined SEER 18 registries and in all geographical regions (Figure 1 and eFigure 1 in the Supplement). In SEER 18, rates increased 46.1% (34.9 [95% CI, 34.1-35.8] to 51.0 [95% CI, 50.0-52.1] per 100 000 population) in this 1-year age transition. The SEER 18 rate ratio incidence increase from 49 to 50 years of age (1.46 [95% CI, 1.42-1.51]) was significantly higher than earlier 1-year age transitions (eTable 1 in the Supplement). Log linear modeling estimated an incidence of 40.7 per 100 000 at 50 years in the theoretical absence of screening, indicating only a 16.6% incidence increase from 49 to 50 years associated with advancing age (eFigure 2 in the Supplement).

Incidence increases from 49 to 50 years ranged from 35.5% in the South (from an incidence of 43.1 [95% CI, 41.1-45.1] per 100 000 at 49 years to an incidence of 58.4 [95% CI, 56.1-60.8] per 100 000 at 50 years) to 56.0% in the Northeast (from an incidence of 34.3 [95% CI, 32.3-36.5] per 100 000 at 49 years to an incidence of 53.5 [95% CI, 50.9-56.2] per 100 000 at 50 years) (eFigure 1 in the Supplement). Steep incidence increases between 49 and 50 years of age were seen for both women (39.1% increase from 32.0 [95% CI, 30.9-33.2] to 44.5 [95% CI, 43.1-45.8] per 100 000) and men (52.9% increase from 37.8 [95% CI, 36.6-39.1] to 57.8 [95% CI, 56.3-59.4] per 100 000) (eFigure 3 in the Supplement). Incidence rate increases from age 49 to 50 years by state, and correlated with previously reported state CRC screening rates, are reported in eTable 2 in the Supplement.^21^ Utah, which had the highest incidence increase from 49 to 50 years of age, had the third highest CRC screening rate for individuals 50 years or older; Connecticut, which had the second highest incidence increase from 49 to 50 years of age, had the highest CRC screening rates for individuals 50 years or older.^21^

Steep incidence increases from 49 to 50 years of age were seen in white populations (46.2% increase from 34.0 [95% CI, 33.0-34.9] to 49.7 [95% CI, 48.6-50.9] per 100 000) and black populations (47.3% increase from 41.9 [95% CI, 39.3-44.7] to 61.7 [95% CI, 58.5-65.1] per 100 000) (eFigure 4 in the Supplement). Steep incidence increases were also seen for colon cancer (51.4% increase from 21.6 [95% CI, 21.0-22.3] to 32.7 [95% CI, 31.9-33.5] per 100 000) and rectal cancer (37.6% increase from 13.3 [95% CI, 12.8-13.8] to 18.3 [95% CI, 17.7-18.9] per 100 000) (eFigure 5 in the Supplement).

**Figure 2.**
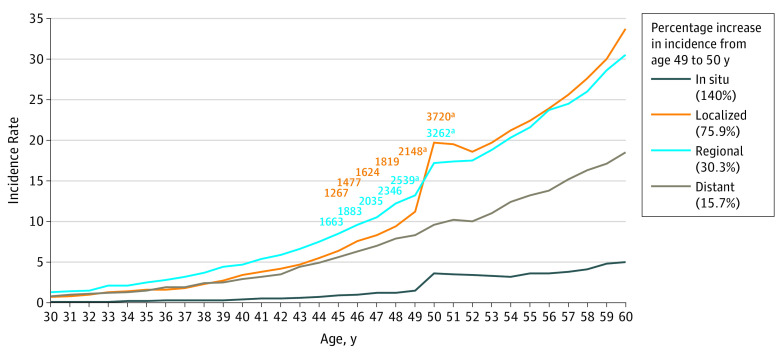
Colorectal Cancer Incidence Rates per 100 000 Population Stratified by Stage in US Surveillance, Epidemiology, and End Results 18 Registries Among Patients Aged 30 to 60 Years, 2000-2015 Within the figure key, the percentage increase in incidence from age 49 to 50 years for each stage is reported. The orange numbers indicate yearly case counts from 45 to 50 years of age in localized stages, and the blue numbers indicate yearly case counts from 45 to 50 years of age in regional stages. ^a^Case counts for 49 and 50 years of age specifically.

Stage stratification showed steep increases in the rate of colorectal cancer at the transition between 49 and 50 years in SEER 18 registry data for localized CRCs (75.9% increase from 11.2 [95% CI, 10.7-11.7] to 19.7 [95% CI, 19.0-20.3] per 100 000) and regional CRCs (30.3% increase from 13.2 [95% CI, 12.7-13.8] to 17.2 [95% CI, 16.7-17.8] per 100 000) compared with other age transitions (Figure 2). Similar incidence rate increases at the transition between 49 and 50 years for localized and regional CRCs were also seen in both the white and black populations (eFigure 6 and eFigure 7 in the Supplement). The rate increase at the transition between 49 and 50 years for distant stage cancers was lower (15.7% increase from 8.3 [95% CI, 7.9-8.7] to 9.6 [95% CI, 9.2-10.1] per 100 000). There was a trend toward a steeper incidence increase in distant CRCs in the black population (27.1%) compared with the white population (13.9%); however, this increase was not significant (rate ratio, 1.27 [95% CI, 1.08-1.50] in the black population vs 1.14 [95% CI, 1.06-1.24] in the white population). Although steep in situ rate increases were seen, rates were low overall compared with invasive cases.

Steep rate increases in localized cancers at the transition between 49 and 50 years were seen in colon cancer (91.0% increase from 6.7 [95% CI, 6.3-7.1] to 12.8 [95% CI, 12.3-13.3] per 100 000) and rectal cancer (53.3% increase from 4.5 [95% CI, 4.2-4.8] to 6.9 [95% CI, 6.5-7.3] per 100 000) (eFigure 8 and eFigure 9 in the Supplement). Steep incidence increases in regional cancers between 49 and 50 years were also seen in colon cancer (31.3% increase from 8.0 [95% CI, 7.6-8.5] to 10.5 [95% CI, 10.1-11.0] per 100 000) and rectal cancer (28.9% increase from 5.2 [95% CI, 4.9-5.5] to 6.7 [95% CI, 6.4-7.1] per 100 000).

Absolute SEER 18 case counts of CRC at each age and stage stratified are shown in Table 2.^22^ There were 2295 additional localized or regional CRC cases in SEER 18 from 49 to 50 years of age alone. At 50 years specifically, 9474 cases of CRC were diagnosed from 2000 to 2015, of which 8799 (92.9%) were invasive. Extrapolating to all 50 states, we estimate that 33 822 CRC cases of any disease stage were diagnosed at 50 years alone. Combining all ages from 45 to 50 years, we found that there were 36 198 cases of CRC in SEER 18 from 2000 to 2015, of which 34 412 (95.1%) were invasive. Extrapolating to all 50 states, there were approximately 129 226 CRCs of any stage from patients aged 45 to 50 years from 2000 to 2015.

**Table 2.** Absolute CRC Case Counts Diagnosed at Each Age From 45 to 50 Years of Age Stratified by Stage in SEER 18 Registries and Estimated Extrapolation of CRC Case Counts to All 50 States^a^

Characteristic	No. by Age, y						
	45	46	47	48	49	50	45-50
In situ CRC case counts in SEER 18 (2000-2015)	177	197	226	227	284	675	1786
Localized CRC case counts in SEER 18 (2000-2015)	1267	1477	1624	1819	2148	3720	12 055
Regional CRC case counts in SEER 18 (2000-2015)	1663	1883	2035	2346	2539	3262	13 728
Distant CRC case counts in SEER 18 (2000-2015)	1097	1239	1363	1521	1592	1817	8629
Total CRC case counts in SEER 18 (2000-2015)	4204	4796	5248	5913	6563	9474	36 198
Total invasive CRC case counts in SEER 18 (2000-2015)^b^	4027	4599	5022	5686	6279	8799	34 412
Invasive CRC case counts per calendar year in SEER 18	268	307	335	379	419	587	2294
Total CRCs extrapolated to all 50 states	15 008	17 122	18 735	21 109	23 430	33 822	129 226
Total invasive CRC case counts extrapolated to all 50 states	14 376	16 418	17 928	20 299	22 416	31 412	122 849

Abbreviations: CRC, colorectal cancer; SEER 18, Surveillance, Epidemiology, and End Results 18 registries.

^a^ Because SEER 18 represents 28% of the US population, to estimate extrapolated CRC case counts to all 50 states, a multiplication factor of 3.57 was used. The SEER data may underestimate national colorectal cancer incidence rates.^22^

^b^ Beyond in situ stage.

**Figure 3.**
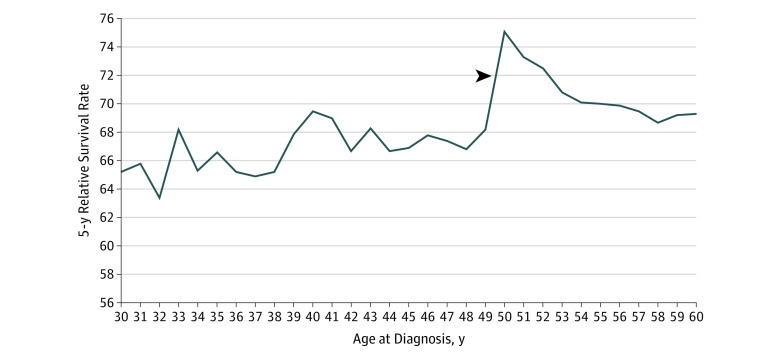
Five-Year Relative Survival by Age in the US Surveillance, Epidemiology, and End Results 18 Registries Among Patients With Colorectal Cancer Aged 30 to 60 Years, 2000-2015 The only statistically significant difference in yearly age transition was from 49 to 50 years. The arrowhead indicates a statistically significant increase in survival from 49 to 50 years of age (absolute increase, 6.9%; relative increase, 10.1%).

In terms of 5-year relative survival, we found a significant 6.9% absolute increase (*P* < .001; relative increase, 10.1%) in survival from 49 to 50 years (5-year survival, 68.2% [95% CI, 66.8%-69.5%] at 49 years to 75.1% [95% CI, 74.0%-76.1%] at 50 years) (Figure 3).

## Discussion

Analysis of CRC incidence in 1-year age increments uncovered steep rate increases (46.1% in SEER 18) from 49 to 50 years of age, with 92.9% of cases being invasive (beyond in situ). These findings suggest a high case burden of preclinical, undetected EOCRC in younger patients (ultimately diagnosed via screening uptake at 50 years) that is not reflected in observed SEER incidence rates. Hence, we cannot reliably compare CRC incidence rates between individuals younger than 50 years and those 50 years or older to estimate effects of earlier screening.

It is likely that the steep increase between ages of 49 and 50 years predominantly reflects increased incidence owing to screening detection as opposed to advancing age. This increase was seen across geographical regions, in men and women, in white and black populations, and in colon and rectal cancer. Screening at 50 years of age unifies these disparate populations. In addition, when we compared our incidence rate data with known colorectal cancer screening rates, Utah, which had the highest incidence increase from 49 to 50 years, had the third highest CRC screening rate for individuals 50 years or older^21^; Connecticut, which had the second highest incidence increase from 49 to 50 years, had the highest CRC screening rates for those 50 years or older (eTable 2 in the Supplement).^21^ However, the association between screening rates and the incidence increase from 49 to 50 years has limitations given that screening rates are reported from 2014 (not 2000-2015) and that screening rates at age 50 years specifically are not available. Rate ratio analysis revealed that the incidence increase from 49 to 50 years was significantly higher than earlier 1-year age transitions when average-risk screening would not be recommended, consistent with this increase being primarily associated with screening detection. Finally, log linear modeling estimated only a 16.6% incidence increase from 49 to 50 years associated with advancing age.

This incidence increase from 49 to 50 years was not seen in prior studies because age-group ranges or blocks, not yearly-age increments, were analyzed, concealing this finding. Because, to our knowledge, there have been no prior oncology studies performing a similar yearly-age analysis, there is no comparison group to place in context the degree of the incidence increase from age 49 to 50 years and the stage distributions at 50 years. However, the steep rate increase with 92.9% invasive cases is undesirable because the ideal goal of screening is to prevent malignant neoplasms by the removal of precancerous polyps.

The stabilization of the incidence increase, seen from 51 to 52 years of age in SEER 18 (Figure 1), may have several potential explanations. Owing to a large number of cancers detected at 50 years of age from first-time screening, the incidence among those at 51 or 52 years of age may be lower than expected because cancers detected at age 50 years of age would no longer present at later ages. Another potential factor is that, given that guidelines explicitly state that average-risk screening should be performed at 50 years, it is plausible that the largest number of patients initiate screening at that age. There may be underlying age-related incidence increases at 51 and 52 years that are masked, possibly owing to relatively lower screening rates compared with at 50 years.

Pertaining to stage stratification, numerous invasive cases (2295 additional localized or regional cases in SEER 18 from 49 to 50 years alone; Figure 2) support the idea that cancers were developing several years before the age of 50 years owing to delayed diagnosis. A rectal cancer study estimates that the age of such cancers at diagnosis is approximately 3 to 4 years.^23^ Doubling times for CRC are estimated to be approximately 1000 days,^24^ which reinforces that many cancers detected at 50 years were present for several years prior.

A component of length time bias, when slower-growing and potentially less clinically significant cancers are detected with screening, may be present.^25^ However, this possibility may be tempered by the findings of steep increases from 49 to 50 years of CRCs associated with morbidity and mortality. Regional CRCs have a 29% to 30% 5-year mortality and may require surgery and chemotherapy.^26^ Localized CRCs have a 10% to 11% 5-year mortality, and most require surgery.^26,27^ Localized and regional CRCs may be associated with psychological distress, economic burden, and impaired quality of life.^28,29^ A study on just the first year after CRC diagnosis revealed mean health care costs of $49 189 for stage 1 cancer, $66 613 for stage 2, $83 980 for stage 3, and $108 599 for stage 4.^30^

In contrast to localized and regional CRCs, it was expected that distant CRCs would not have steep rate increases from 49 to 50 years of age because these CRCs would more likely present with symptoms prior to screening.^31^ With regard to tumor location, the incidence increase for localized colon cancer nearly doubled from 49 to 50 years (91.0% increase) and was higher than the incidence increase in rectal cancer (53.3%). This finding may be due to rectal cancers being detected prior to 50 years of age because of symptoms.^32^

In terms of 5-year relative survival, given that a key goal of screening is to prolong life expectancy, the 6.9% absolute survival increase and 10.1% relative survival increase from 49 to 50 years further support that incidence increases from 49 to 50 years may be associated with screening uptake. In addition, this finding provides population-based data on the potential beneficial outcomes of CRC screening. However, there may be a component of lead-time bias that may underlie this increase in 5-year survival owing to earlier diagnosis of lesions from screening. As can be seen in Figure 3, there is a decrease in 5-year survival after 50 years, although rates remain high compared with rates among those younger than 50 years. A possible explanation for this declining survival after age 50 years may be lower treatment rates among older patients compared with younger patients.^33^

Our findings contribute to the debates about earlier CRC screening. Despite increasing EOCRC rates and presentations with advanced-stage disease, possible reasons posed against earlier screening include an absolute incidence among patients aged 45 to 49 years that is considered relatively low and potentially diverting resources from higher-risk older patients with nonoptimized screening rates.^1,34^ Our findings, however, suggest the presence of a high case burden of undetected preclinical EOCRC in younger patients, not reflected in observed SEER incidence rates. Hence, the overall underlying EOCRC burden (combined detected and undetected cases) for individuals aged 45 to 49 years is underestimated and may approach that of individuals in their early 50s. It has been postulated that the near doubling of incidence from those aged 45 to 49 years (34 per 100 000) to those aged 50 to 54 years (60.2 per 100 000) is a potential rationale that screening for 45- to 49-year-old patients may be less effective.^35^ To the contrary, our data support that the incidence of CRC increases substantially among individuals in their early 50s compared with individuals in their late 40s, not because rates are truly lower among those aged 45 to 49 years but because CRCs are present but undetected until diagnosed at 50 years when screening is ultimately initiated.

Analyzing absolute case counts helps place these incidence rates in a better perspective. We estimate that at 50 years of age alone, there were approximately 33 822 CRC cases diagnosed from 2000 to 2015 in the United States and approximately 129 226 cases from 45 to 50 years of age (approximately 43 075 cases during a 5-year calendar period). Because most of these cases cluster in individuals in their late 40s, most could be prevented, or at least detected at a lower stage, with earlier screening at 45 years.

A recent study used modeling to estimate that 29 400 cases of CRC could be prevented during the next 5 years with screening at 45 years of age but that it would cost an incremental $10.4 billion, which is thought to be likely cost-effective.^34^ This modeling took into account the potential presence of undetected cases. Our findings provide real-world SEER 18 incidence data, adding to these modeling estimations. In terms of the 43 075 cases during a 5-year calendar period that we estimate among individuals aged 45 to 50 years, it is difficult to predict what percentage could be altogether prevented, as opposed to diagnosed at an earlier stage. However, in addition to prevention, diagnosing some cancers at an earlier stage could potentially be associated with more favorable cost-benefit and resource allocation balances and should be taken into account when estimating the potential effects of a screening threshold at 45 years of age. Furthermore, screening rates are markedly suboptimal among 50- to 54-year-old patients (28%-47% during our study period).^36^ Hence, true CRC case counts at age 50 years (and thus cancers that could be prevented with earlier screening) are likely considerably higher than those observed in our study, which could shift financial and resource-related balances. Further study of previously described hybrid strategies in which stool-based testing may be used for younger, average-risk patients with transition to colonoscopy at older ages may provide a better understanding of cost-benefit balances.^34^

### Limitations and Strengths

This study has some limitations, including its ecologic design. Hence, although we can infer that steep CRC incidence increases from 49 to 50 years of age are due to the increased detection of potentially asymptomatic preclinical CRCs, this possibility cannot be confirmed. Nevertheless, even though there are no specific outcomes data, we performed detailed stage analyses. The 92.9% rate of invasive cases supports the finding that almost all CRCs accounted for in the incidence increase from 49 to 50 years, whether detected via screening or diagnostic testing, require aggressive treatment (surgery and/or chemotherapy and/or radiotherapy). Furthermore, it is not possible to definitively determine the number of years that CRCs began growing prior to being diagnosed at 50 years. Also, SEER 18 represents 28% of the US population, potentially impeding extrapolation to other US areas, including our estimation of CRC case counts nationwide. However, it is believed that SEER may underestimate CRC incidence and mortality compared with combined US data.^22^

This study also has some strengths, including a large study population (170 434 cases) over a 15-year period. More important, a detailed yearly age incidence assessment analyzing transitions from 49 to 50 years of age has never been performed, to our knowledge. The American Cancer Society discussed the possibility that the increased incidence among 50- to 54-year-old patients may be associated with advancing age as well as screening uptake and suggested that the true underlying risk in younger patients may be higher than age rates would indicate.^2^ Our analysis of the transition from 49 to 50 years provides new, registry-based data regarding risk among individuals younger than 50 years, which can add to preexisting modeling studies to help inform decision-making on the age at which to initiate screening. Furthermore, preexisting EOCRC ecologic studies have been broad in scope, focusing on increasing incidence (annual percentage change) in wide, age-range blocks as opposed to providing detailed information on case counts and cancer stage distributions among those approaching or at the screening age of 50 years. Finally, the demonstration of a significant increase in survival from 49 to 50 years of age provides population-based data that support the merits of CRC screening.

## Conclusions

Steep increases in the incidence of invasive, beyond in situ cancers from ages 49 to 50 years are consistent with a high case burden of preclinical CRCs that were undetected in younger patients before the patients received a diagnosis via screening uptake at 50 years. These cancers were not reflected in observed SEER rates among individuals younger than 50 years; therefore, relying on SEER incidence rates among individuals aged 45 to 49 years alone to estimate potential outcomes of earlier screening at 45 years may underestimate the number of CRCs that can be prevented or diagnosed at earlier stages.

More detailed studies are required to determine what proportion of CRC cases are diagnosed at 50 years through screening vs diagnostic testing. Modeling studies, incorporating the steep incidence inflection point at 49 to 50 years, can also be conducted to estimate what the incidence rate increase at 45 years would be with earlier screening. Such studies can also focus on cost-benefit analysis, quality-adjusted life-years, and other metrics to better understand the effects of earlier screening. Finally, a similar 1-year increment SEER analysis could be used to study other cancers subject to screening protocols, including breast and prostate cancers.

## Notes

Supplement. **eTable 1.** Percent Increase and Rate Ratio Analysis for Colorectal Cancer Incidence Rates at Ages 47-48, 48-49, and 49-50 in US SEER^ 18 and US Regions **eTable 2.** Incidence Rate Increase From Age 49 to 50 and Colorectal Cancer Screening Rate by State **eFigure 1.** 2000-2015 Colorectal Cancer Incidence Rates per 100 000 in One Year Age Increments for United States Regions (West, South, Northeast, Midwest) Age 30-60 **eFigure 2.** Log-Linear Model Based on Yearly-Age Incidence Changes at Ages 30-49 to Estimate Colorectal Cancer Incidence at Age 50 in the Theoretical Absence of Screening **eFigure 3.** 2000-2015 Colorectal Cancer Incidence Rates per 100 000 in One Year Age Increments Stratified by Sex, Age 30-60 **eFigure 4.** 2000-2015 Colorectal Cancer Incidence Rates per 100 000 Stratified by Race in Whites Versus Blacks in United States SEER^ 18, Age 30-60 **eFigure 5.** 2000-2015 Colorectal Cancer Incidence Rates per 100 000 Stratified by Location (Colon and Rectum) in United States SEER^ 18, Age 30-60 **eFigure 6.** 2000-2015 Colorectal Cancer Incidence Rates per 100 000 Stratified by Stage in Whites in United States Surveillance, Epidemiology, and End Results Program (SEER) 18, Age 30-60 **eFigure 7.** 2000-2015 Colorectal Cancer Incidence Rates per 100 000 Stratified by Stage in Blacks in United States Surveillance, Epidemiology, and End Results Program (SEER) 18, Age 30-60 **eFigure 8.** 2000-2015 Colon Cancer Incidence Rates per 100 000 Stratified by Stage in United States Surveillance, Epidemiology, and End Results (SEER) Program, Age 30-60 **eFigure 9.** 2000-2015 Rectal Cancer Incidence Rates per 100 000 Stratified by Stage in United States Surveillance, Epidemiology, and End Results (SEER) Program, Age 30-60
