# Errors in Results

**DOI:** 10.1001/jamanetworkopen.2020.1038

**Published:** 2020-02-19

In the Original Investigation titled “Trends in Incidence of Early-Onset Colorectal Cancer in the United States Among Those Approaching Screening Age,”^1^ published January 31, 2020, there was an error in the data given in the Results section of the abstract and 2 errors in the data given in the Results section of the text. The lower bound of the 95% CI for the rate ratio incidence increase in the Surveillance, Epidemiology, and End Results 18 registries from 49 to 50 years of age should be changed from 1.43 to 1.42 in the Results section of the abstract and the text. The percentage of black patients in the Results section of the text should be changed from 14.4% to 14.3%. This article has been corrected.^1^
