# Supplementary material for: Trends in Incidence of Early-Onset Colorectal Cancer in the United States Among Those Approaching Screening Age
Source: JAMA Netw Open. 2020 Jan 31;3(1):e1920407. doi: 10.1001/jamanetworkopen.2019.20407 (PMC7042874; doi:10.1001/jamanetworkopen.2019.20407)
Supplement: Supplement. — eTable 1. Percent Increase and Rate Ratio Analysis for Colorectal Cancer Incidence Rates at Ages 47-48, 48-49, and 49-50 in US SEER^ 18 and US Regions eTable 2. Incidence Rate Increase From Age 49 to 50 and Colorectal Cancer Screening Rate by State eFigure 1. 2000-2015 Colorectal Cancer Incidence Rates per 100 000 in One Year Age Increments for United States Regions (West, South, Northeast, Midwest) Age 30-60 eFigure 2. Log-Linear Model Based on Yearly-Age Incidence Changes at Ages 30-49 to Estimate Colorectal Cancer Incidence at Age 50 in the Theoretical Absence of Screening eFigure 3. 2000-2015 Colorectal Cancer Incidence Rates per 100 000 in One Year Age Increments Stratified by Sex, Age 30-60 eFigure 4. 2000-2015 Colorectal Cancer Incidence Rates per 100 000 Stratified by Race in Whites Versus Blacks in United States SEER^ 18, Age 30-60 eFigure 5. 2000-2015 Colorectal Cancer Incidence Rates per 100 000 Stratified by Location (Colon and Rectum) in United States SEER^ 18, Age 30-60 eFigure 6. 2000-2015 Colorectal Cancer Incidence Rates per 100 000 Stratified by Stage in Whites in United States Surveillance, Epidemiology, and End Results Program (SEER) 18, Age 30-60 eFigure 7. 2000-2015 Colorectal Cancer Incidence Rates per 100 000 Stratified by Stage in Blacks in United States Surveillance, Epidemiology, and End Results Program (SEER) 18, Age 30-60 eFigure 8. 2000-2015 Colon Cancer Incidence Rates per 100 000 Stratified by Stage in United States Surveillance, Epidemiology, and End Results (SEER) Program, Age 30-60 eFigure 9. 2000-2015 Rectal Cancer Incidence Rates per 100 000 Stratified by Stage in United States Surveillance, Epidemiology, and End Results (SEER) Program, Age 30-60 [file jamanetwopen-e1920407-s001.pdf]

Supplementary Online Content

Abualkhair WH, Zhou M, Ahnen D, Yu Q, Wu X-C, Karlitz JJ. Trends in incidence of early-onset colorectal cancer in the United States among those approaching screening age. *JAMA Netw Open.* 2020;3(1):e1920407.
doi:10.1001/jamanetworkopen.2019.20407

eTable 1. Percent Increase and Rate Ratio Analysis for Colorectal Cancer Incidence Rates at Ages 47-48, 48-49, and 49-50 in US SEER^ 18 and US Regions
eTable 2. Incidence Rate Increase From Age 49 to 50 and Colorectal Cancer Screening Rate by State
eFigure 1. 2000-2015 Colorectal Cancer Incidence Rates per 100,000 in One Year Age Increments for United States Regions (West, South, Northeast, Midwest) Age 30-60
eFigure 2. Log-Linear Model Based on Yearly-Age Incidence Changes at Ages 30-49 to Estimate Colorectal Cancer Incidence at Age 50 in the Theoretical Absence of Screening
eFigure 3. 2000-2015 Colorectal Cancer Incidence Rates per 100,000 in One Year Age Increments Stratified by Sex, Age 30-60
eFigure 4. 2000-2015 Colorectal Cancer Incidence Rates per 100,000 Stratified by Race in Whites Versus Blacks in United States SEER^ 18, Age 30-60
eFigure 5. 2000-2015 Colorectal Cancer Incidence Rates per 100,000 Stratified by Location (Colon and Rectum) in United States SEER^ 18, Age 30-60
eFigure 6. 2000-2015 Colorectal Cancer Incidence Rates per 100,000 Stratified by Stage in Whites in United States Surveillance, Epidemiology, and End Results Program (SEER) 18, Age 30-60
eFigure 7. 2000-2015 Colorectal Cancer Incidence Rates per 100,000 Stratified by Stage in Blacks in United States Surveillance, Epidemiology, and End Results Program (SEER) 18, Age 30-60
eFigure 8. 2000-2015 Colon Cancer Incidence Rates per 100,000 Stratified by Stage in United States Surveillance, Epidemiology, and End Results (SEER) Program, Age 30-60
eFigure 9. 2000-2015 Rectal Cancer Incidence Rates per 100,000 Stratified by Stage in United States Surveillance, Epidemiology, and End Results (SEER) Program, Age 30-60

This supplementary material has been provided by the authors to give readers additional information about their work.

© 2020 Abualkhair WH et al. *JAMA Network Open.*

eTable 1. Percent Increase and Rate Ratio Analysis for Colorectal Cancer Incidence Rates at Ages 47-48, 48-49, and 49-50 in U.S. SEER^ 18 and U.S. Regions

	SEER 18		West		Northeast		South		Midwest	
	Rate Increase (%)	Rate Ratio (CI)	Rate Increase (%)	Rate Ratio (CI)	Rate Increase (%)	Rate Ratio (CI)	Rate Increase (%)	Rate Ratio (CI)	Rate increase (%)	Rate Ratio (CI)
49-50	34.9 to 51.0 (46.10%)	1.46 (1.42-1.51)	32.0 to 47.3 (47.80%)	1.48 (1.41-1.54)	34.3 to 53.5 (56.00%)	1.56 (1.44-1.69)	43.1 to 58.4 (35.50%)	1.36 (1.27-1.44)	33.7 to 51.8 (53.70%)	1.53 (1.38-1.71)
48-49	31.3 to 34.9 (11.50%)	1.12 (1.08-1.16)	28.4 to 32.0 (12.70%)	1.13 (1.07-1.18)	32.4 to 34.3 (5.90%)	1.06 (0.97-1.16)	37.3 to 43.1 (15.60%)	1.16 (1.08-1.24)	32.5 to 33.7 (3.70%)	1.04 (0.92-1.17)
47-48	27.5-31.3 (13.80%)	1.14 (1.10-1.18)	25.2 to 28.4 (12.70%)	1.13 (1.07-1.19)	27.5 to 32.4 (17.80%)	1.18 (1.07-1.29)	32.5 to 37.3 (14.80%)	1.15 (1.07-1.24)	28.9 to 32.5 (12.50%)	1.12 (0.99-1.27)

Abbreviations: CI, confidence interval are 95% for rates and ratios; U.S. United States

^SEER indicates the Surveillance, Epidemiology, and End Results program

© 2020 Abualkhair WH et al. *JAMA Network Open.*

eTable 2. Incidence Rate Increase From Age 49 to 50 and Colorectal Cancer Screening Rate by State

	Age 49 Incidence Rate	Age 50 Incidence Rate	Percent Increase From Age 49 to 50	Colorectal Screening Rate in 2014 ≥50 Years-Old^
Alaska#	70	106	51.40%	61.20%
California†	32.2	47.1	46.30%	68.60%
Connecticut*	34.4	62.5	81.70%	73.80%
Georgia‡	39.1	56.4	44.20%	67.60%
Hawaii	42	65.9	56.90%	69.30%
Iowa	32.9	55.5	68.70%	68.20%
Kentucky	47.1	61.6	30.80%	68.10%
Louisiana	47.5	59.4	25.10%	65.80%
Michigan&	34.4	49	42.40%	72.10%
New Jersey	34.3	49.7	44.90%	66.40%
New Mexico	31.1	35.4	13.80%	62.50%
Utah*	25.2	49.7	97.20%	70.70%
Washington§	30.5	46.4	52.10%	70.10%

*States with the 1st (Utah) and 2nd (Connecticut) highest incidence rate increases from 49 to 50 had the 3rd and 1st highest CRC screening rates in those 50 and older respectively.
#Alaska includes Alaska natives registry only.
†California includes Los Angeles registry, Greater California registry, San Jose-Monterey registry, and San Francisco-Oakland registry.
‡Georgia includes Atlanta registry, Greater Georgia registry, and Rural Georgia registry.
&Michigan includes Detroit registry only.
§Washington includes Seattle-Puget Sound registry.
^State screening rates in this table are adopted from the American Cancer Society (Reference 21 in the manuscript).

© 2020 Abualkhair WH et al. *JAMA Network Open.*

eFigure 1. 2000-2015 Colorectal Cancer Incidence Rates per 100,000 in One Year Age Increments for United States Regions (West, South, Northeast, Midwest) Age 30-60

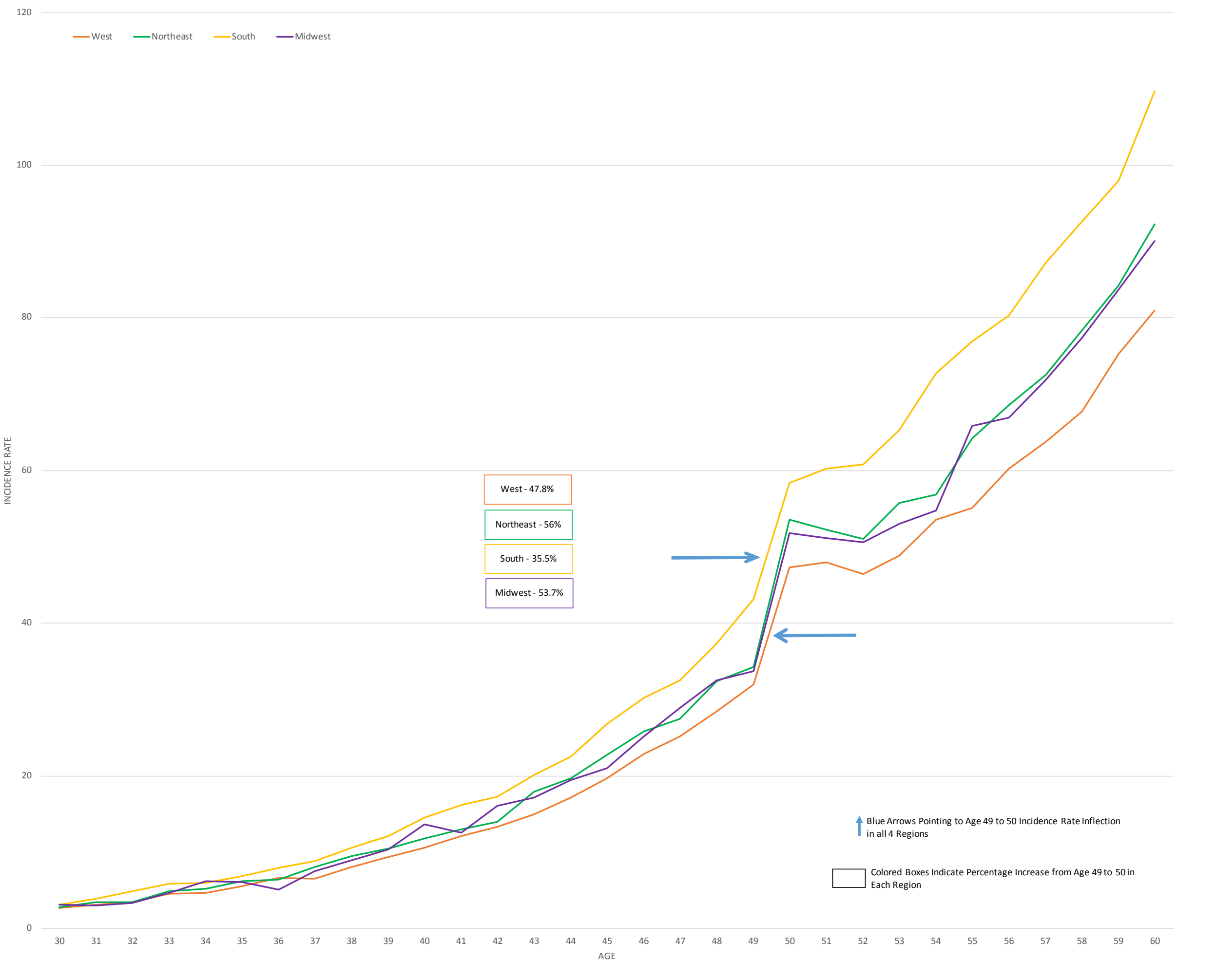

© 2020 Abualkhair WH et al. *JAMA Network Open.*

eFigure 2. Log-Linear Model Based on Yearly-Age Incidence Changes at Ages 30-49 to Estimate Colorectal Cancer Incidence at Age 50 in the Theoretical Absence of Screening

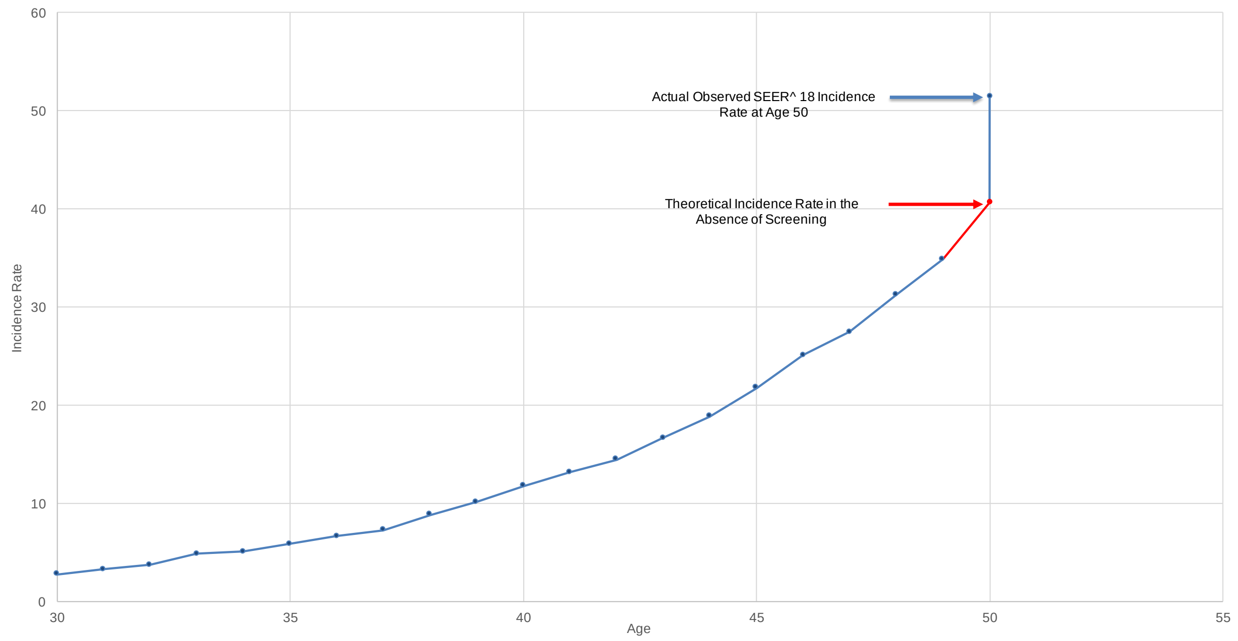

^SEER indicates the Surveillance, Epidemiology, and End Results program

Note. The outcome variable in our study was case count, so we fitted a log-linear model with the total number of populations at each age group as the offset. The analysis was based on the case counts at ages 30-49 to estimate the counts at age 50 in the theoretical absence of screening. The incidence at age 50 was quotient of case count and population.

© 2020 Abualkhair WH et al. *JAMA Network Open.*

eFigure 3. 2000-2015 Colorectal Cancer Incidence Rates per 100,000 in One Year Age Increments Stratified by Sex, Age 30-60

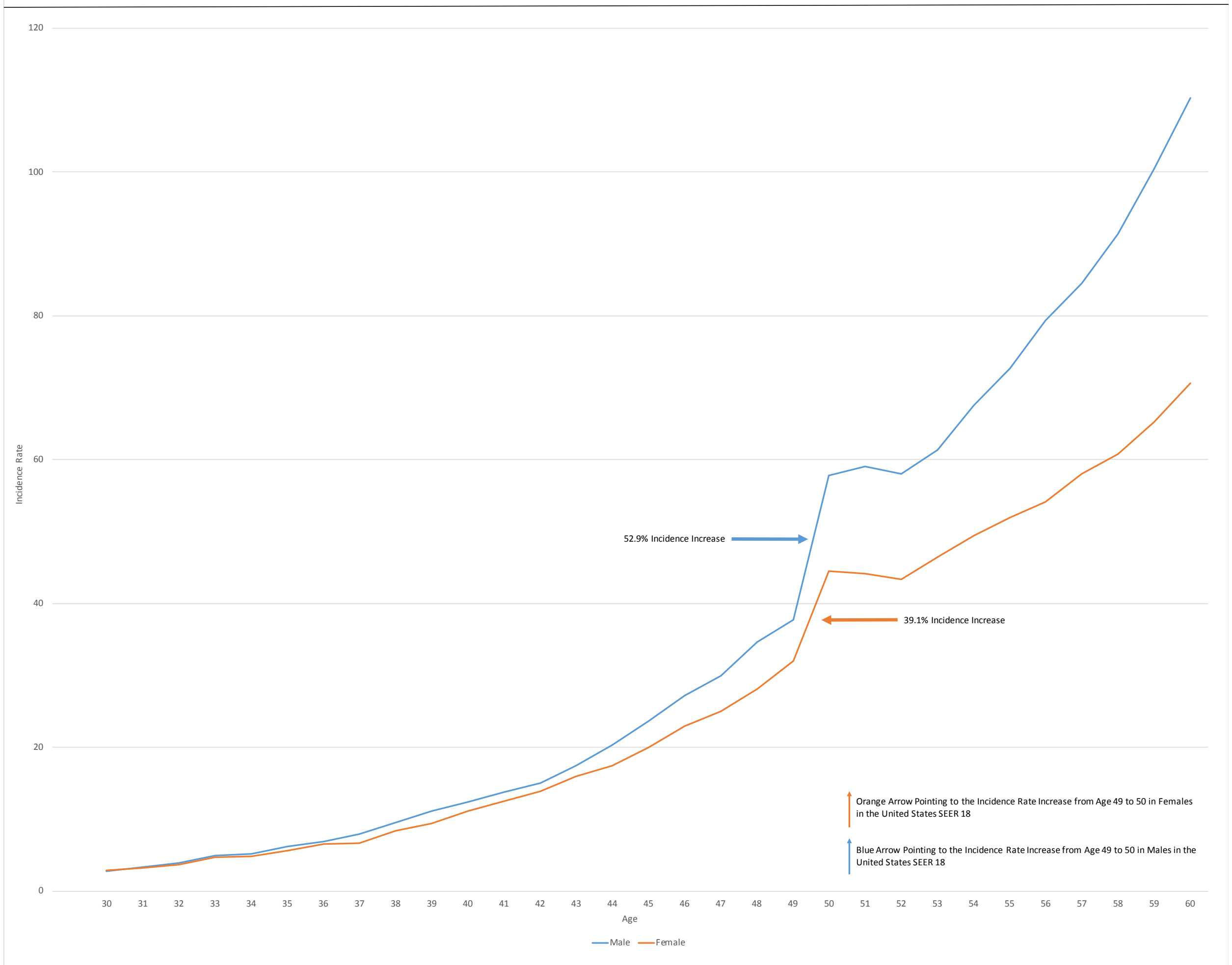

© 2020 Abualkhair WH et al. *JAMA Network Open.*

eFigure 4. 2000-2015 Colorectal Cancer Incidence Rates per 100,000 Stratified by Race in Whites Versus Blacks in United States SEER^ 18, Age 30-60

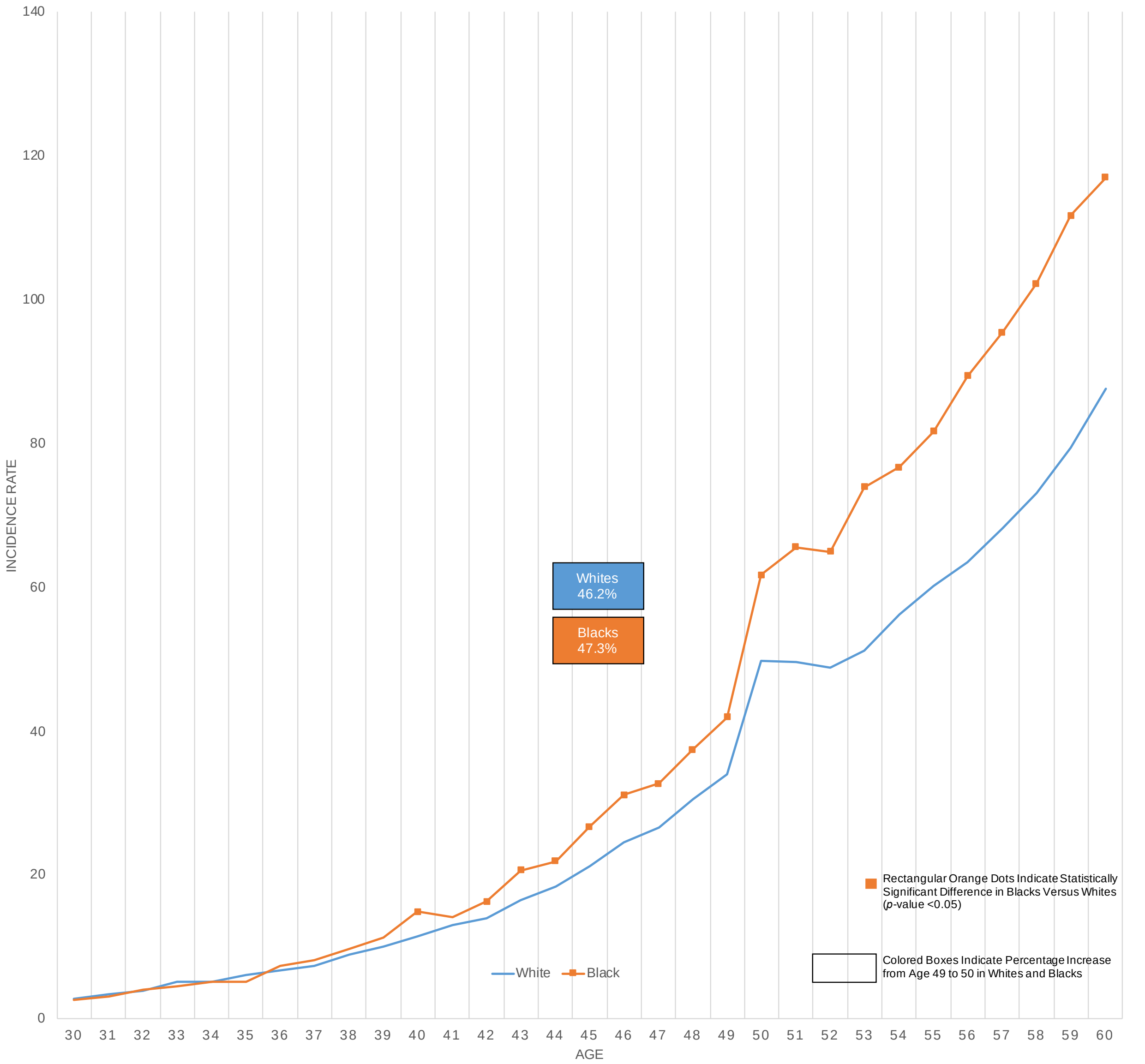

^SEER indicates the Surveillance, Epidemiology, and End Results program

© 2020 Abualkhair WH et al. *JAMA Network Open.*

eFigure 5. 2000-2015 Colorectal Cancer Incidence Rates per 100,000 Stratified by Location (Colon and Rectum) in United States SEER^ 18, Age 30-60

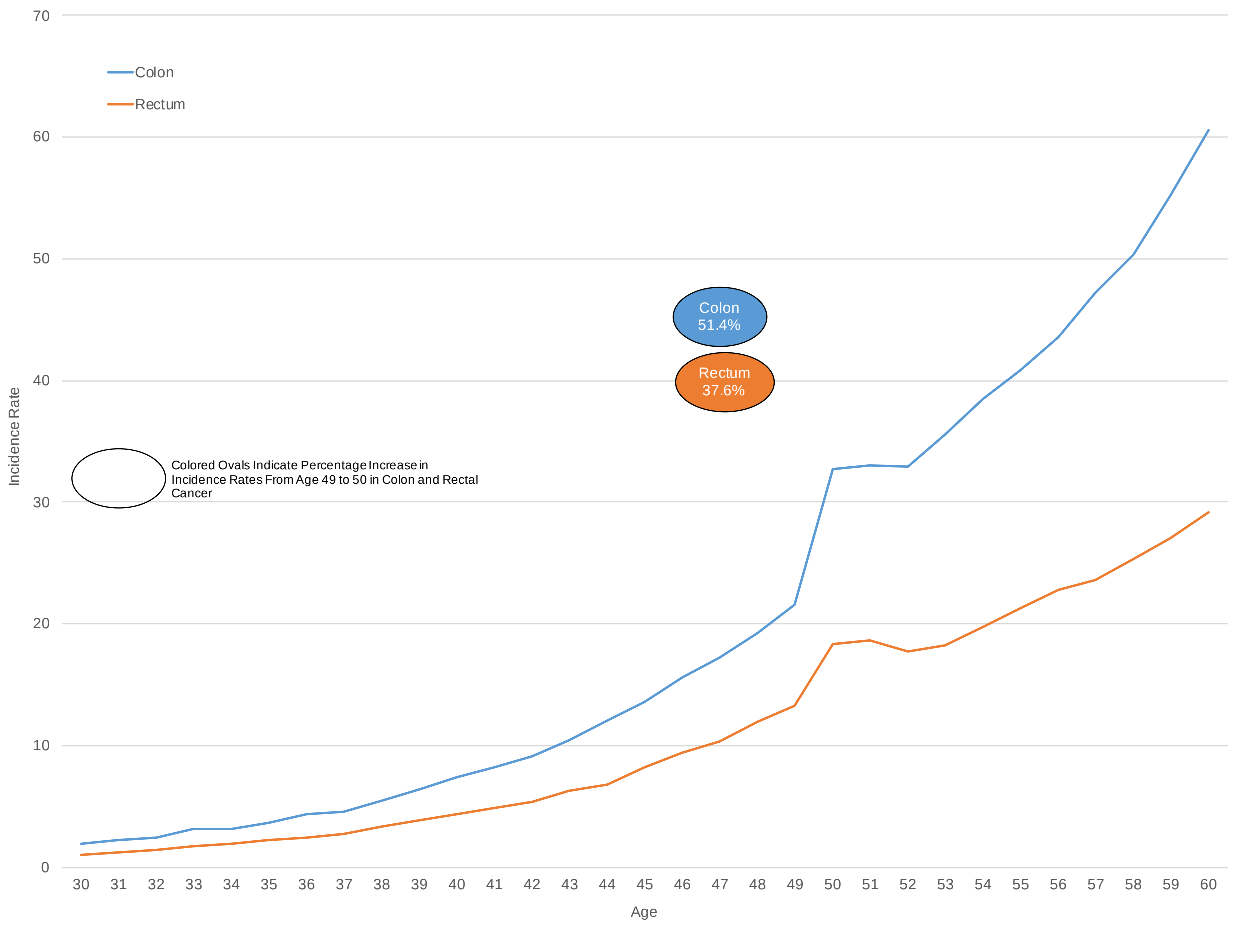

^SEER indicates the Surveillance, Epidemiology, and End Results program

© 2020 Abualkhair WH et al. *JAMA Network Open.*

eFigure 6. 2000-2015 Colorectal Cancer Incidence Rates per 100,000 Stratified by Stage in Whites in United States Surveillance, Epidemiology, and End Results Program (SEER) 18, Age 30-60

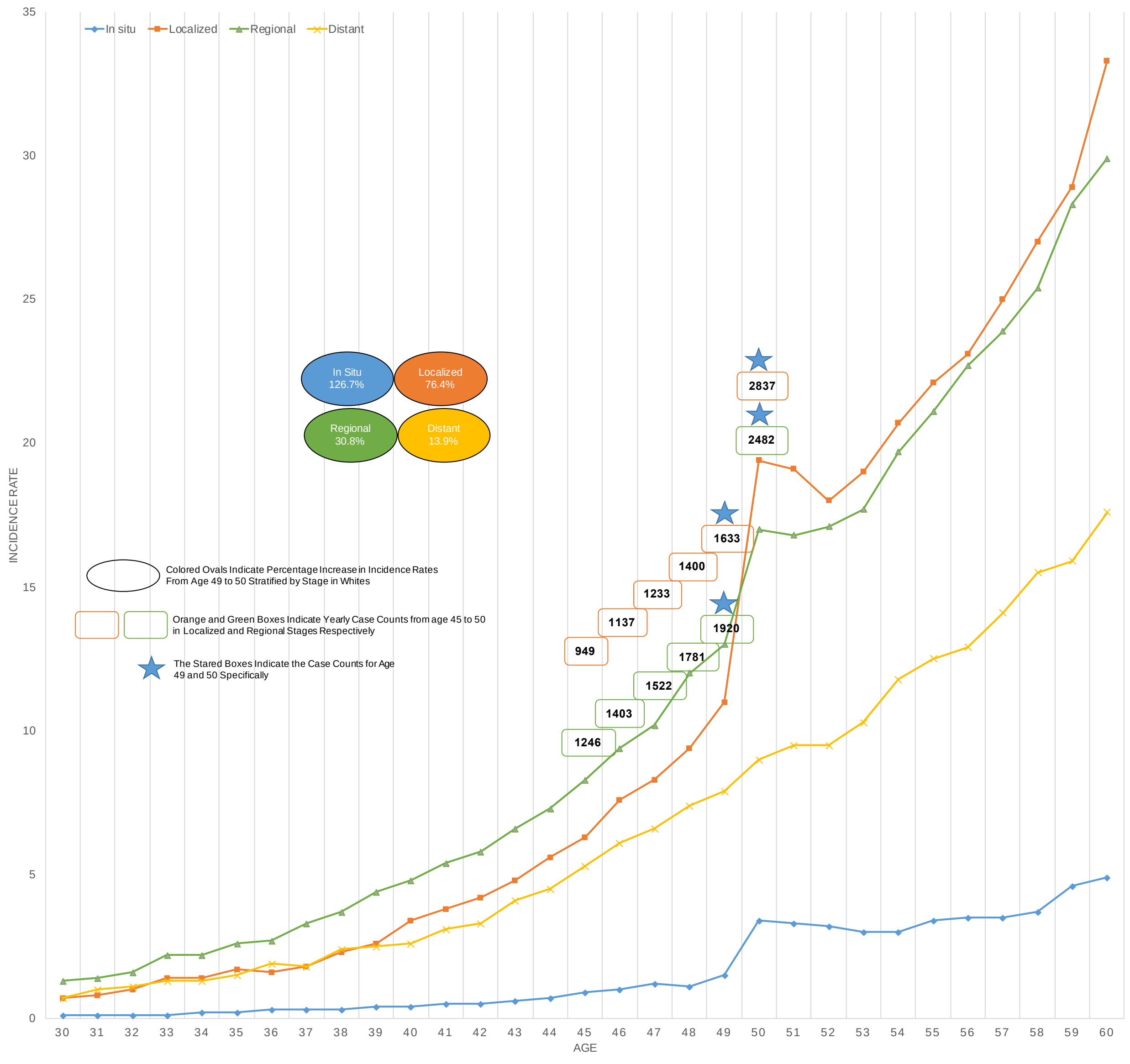

© 2020 Abualkhair WH et al. *JAMA Network Open.*

eFigure 7. 2000-2015 Colorectal Cancer Incidence Rates per 100,000 Stratified by Stage in Blacks in United States Surveillance, Epidemiology, and End Results Program (SEER) 18, Age 30-60

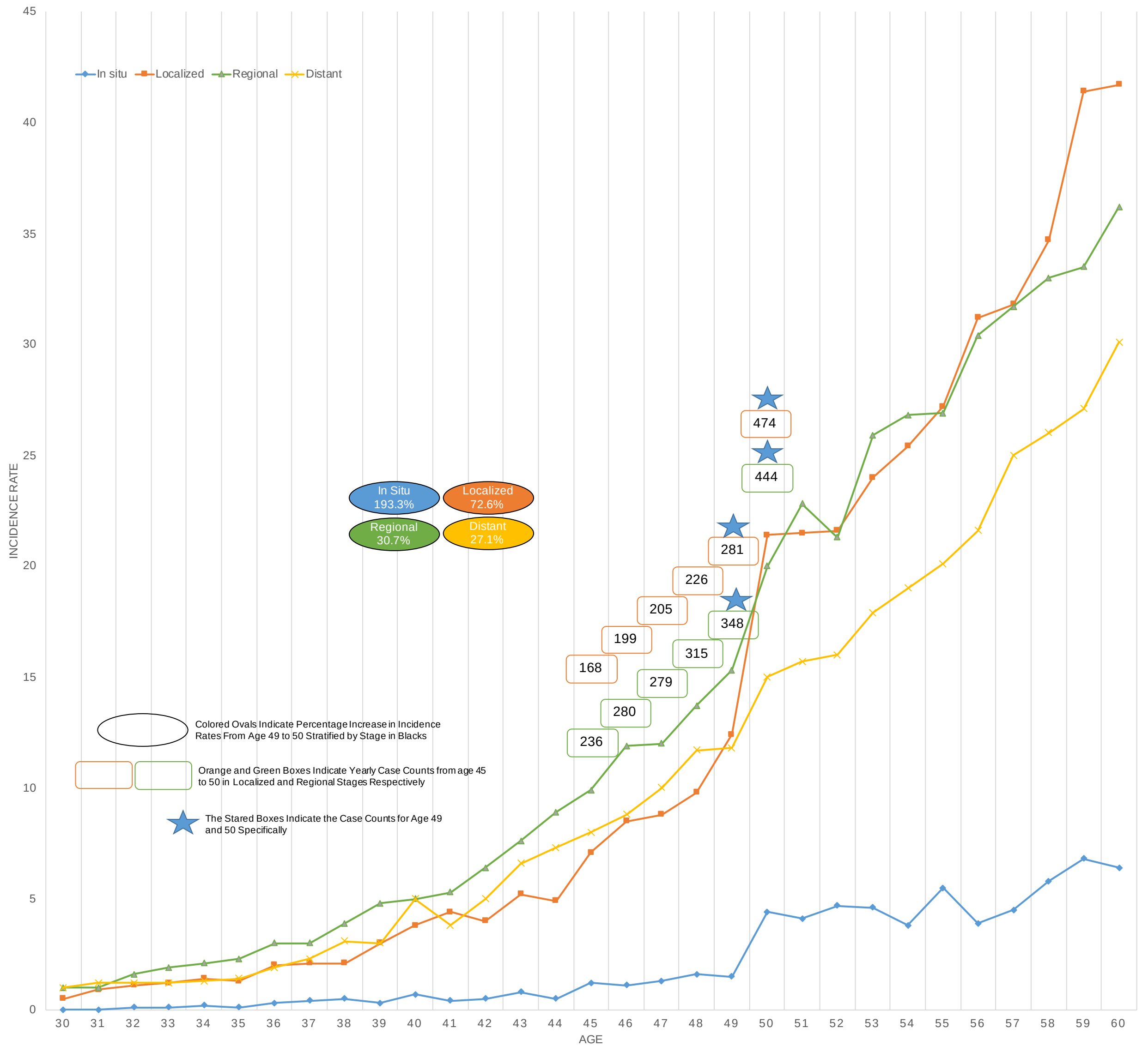

© 2020 Abualkhair WH et al. *JAMA Network Open.*

eFigure 8. 2000-2015 Colon Cancer Incidence Rates per 100,000 Stratified by Stage in United States Surveillance, Epidemiology, and End Results (SEER) Program, Age 30-60

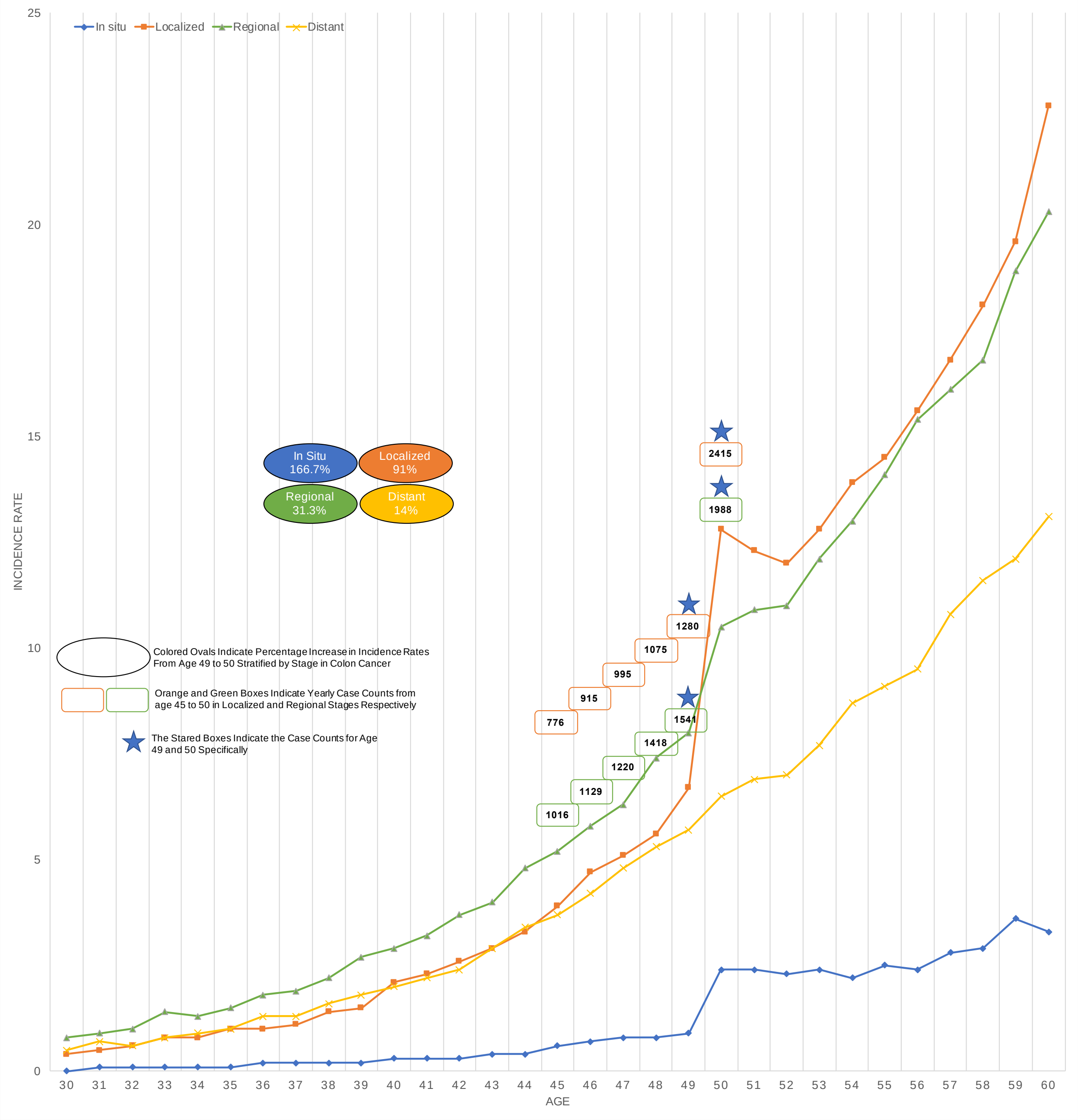

© 2020 Abualkhair WH et al. *JAMA Network Open.*

eFigure 9. 2000-2015 Rectal Cancer Incidence Rates per 100,000 Stratified by Stage in United States Surveillance, Epidemiology, and End Results (SEER) Program, Age 30-60

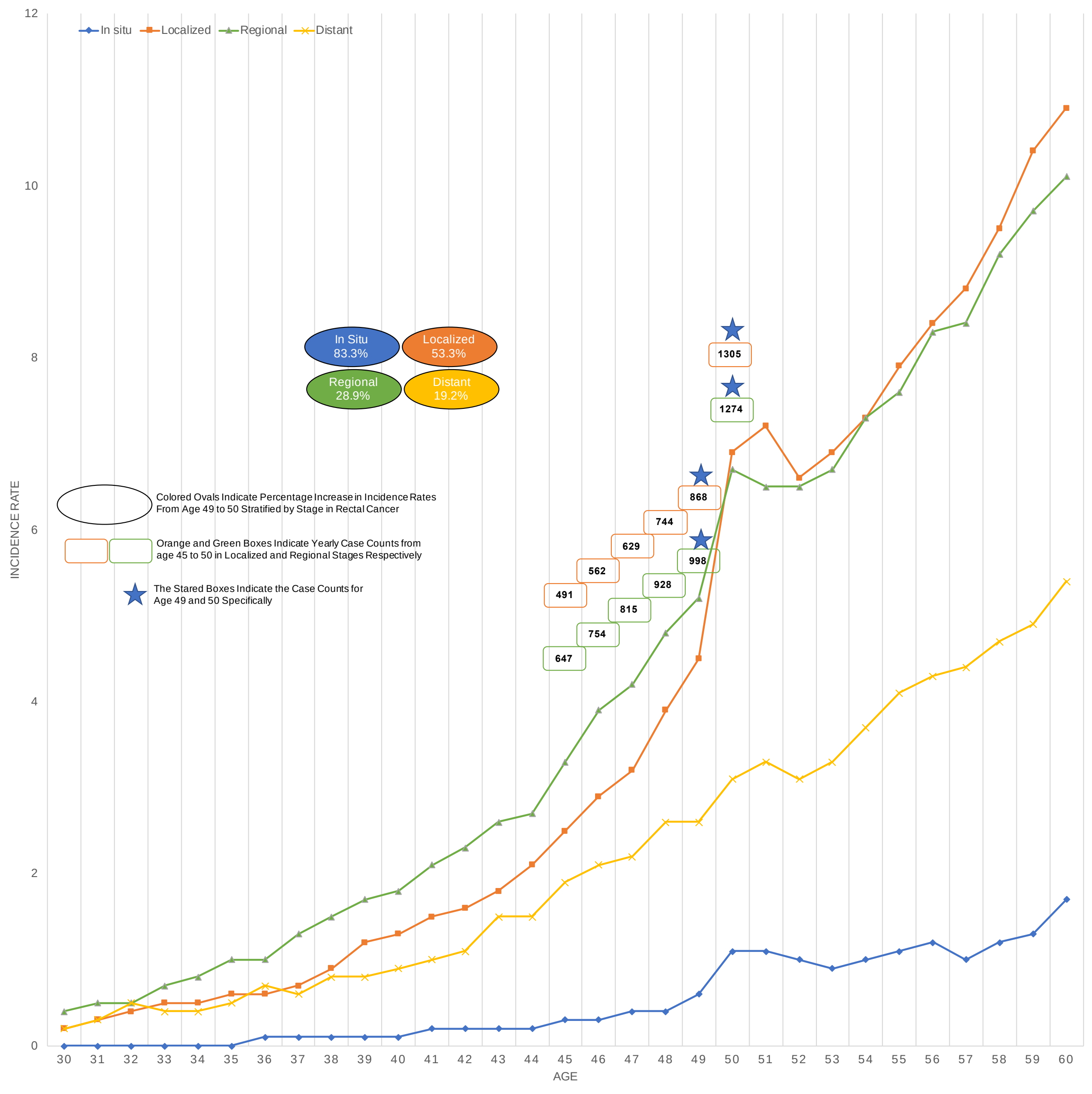

© 2020 Abualkhair WH et al. *JAMA Network Open.*
